# Evaluation of chromosomal aberrations caused by air pollutants in some taxi drivers from two polluted districts of urban Tehran and its comparison with drivers from rural areas of Lahijan: a pilot study

**DOI:** 10.1186/s40201-014-0144-0

**Published:** 2014-12-19

**Authors:** Sara Taghizadeh, Hossein Najmabadi, Koorosh Kamali, Farkhondeh Behjati

**Affiliations:** Genetics Research Center, University of Social Welfare and Rehabilitation Sciences, Tehran, Iran; Department of Public Health, School of Public Health, Zanjan University of Medical Sciences, Zanjan, Iran

**Keywords:** Air pollution, Chromosome aberration, Taxi drivers, Tehran, Lahijan

## Abstract

**Background:**

Chromosome instability is the most common form of genomic instability. Genomic instability can lead to tumorogenesis. High level of chromosomal aberrations in peripheral blood lymphocytes can be used as a biomarker for cancer. Air pollution is one of the most important factors that cause chromosomal instability (CIN). In this comparative study we used classic Cytogenetic technique to analyze the effects of air pollutants on chromosome stability. We collected peripheral blood from 30 taxi drivers of two polluted districts (districts 6 and 7) in Tehran and 30 taxi drivers from rural areas of Lahijan, north of Iran.

**Results:**

Comparison of the level of chromosome breakage in the two groups showed an increased level of chromosome breakage in the drivers from polluted districts of Tehran, although not significant, using Fisher exact test (p-value = 0.300). However, the overall chromosome aberration rate (including both chromosome and chromatid gaps), the difference was significant using Chi-square test (p-value = 0.012).

**Conclusion:**

An increased level of chromosome aberration was present in the drivers from polluted districts of Tehran compared to drivers from non-polluted areas in Lahijan.

## Introduction

Air pollution is concerned with many ill effects on health including pulmonary and cardiovascular diseases, cancer as well as increased mortality [1]. Additionally, a relationship between intrauterine growth retardation and high exposure to air pollutants during pregnancy has been reported [2]. This has been demonstrated in children and pregnant mothers living in industrial regions. Chromosomal aberrations and gene mutations lead to different kinds of diseases including cancer, particularly Leukemia [3]. The damaging pollutants include poly aromatic hydrocarbons (PAH), methylbenzene, inhaled Ozone, stress oxidative damage of oxygen (ROS), nitrogen (RNS), and persistent particulate matter with size less than 2.5 micron [4].

PAHs are widespread environmental toxicants in ambient air produced from the partial combustion of fossil fuels. Different studies have evaluated the genetic damage in residents in polluted cities and compared them with those living in rural areas. They have shown that air pollution can have carcinogenic effect causing chromosomal aberrations in adults [5].

Adverse health effects are inflicted through physical and chemical properties of air born particulate matter (PM). These pollutants may induce either direct or indirect damage to biological macromolecules, including DNA. Several studies have indicated that pollutants have destructive effect on chromosomes and other important biomolecules [6]. There are both exogenous physical agents and endogenous chemical genotoxic agents, both of which cause DNA damage including double strand and single strand breaks. Besides, ultraviolet can induce both interstrand and intrastrand crosslinks. They can all contribute towards formation of DNA adducts. Defect in repair and replication mechanisms are two outcomes of DNA damage. Such DNA defects can cause mutation or chromosomal aberrations leading to genomic instability [7]. The common damages on chromosomes are breaks, gaps, translocations and acentric fragments [8].

Genomic instability is recognized as a major stimulating force of tumorogenesis [9]. The final goal of cell division for most non-cancerous somatic cells is to accurately duplicate the genome and then evenly divide into the two daughter cells. However any deregulation of cell cycle can lead to aberrant cells. Accumulation of these genetic alterations due to failure in regulation of cell division, the imbalance between cell growth and death can eventually lead to cancer [10]. There are different forms of genomic instability. The most common form is called chromosomal instability (CIN), leading to high rate of changes in chromosome structure and number, which were first observed more than a hundred years ago [11].

The accepted technique for biological monitoring of genetic damage to somatic cells is conventional cytogenetic analysis of peripheral blood lymphocytes, which has been adapted internationally since 1970. It has the advantage of determining the DNA damage by physical and chemical clastogenic agents in the occupational situation. Today, chromosomal aberration in human peripheral lymphocytes is recognized as a valuable biomarker of effect. It is generally accepted that a high frequency of chromosomal aberrations in peripheral lymphocytes is predictive of an increased risk of cancer [12].

### The air pollution in Tehran

**Figure 1:**
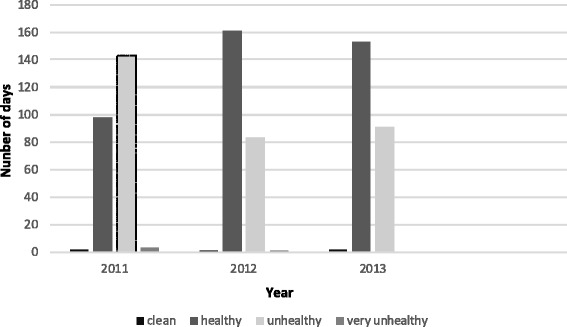
**Comparison of the level of air pollution from March to October [** 14 **].**

The world Health organization (WHO) was warned about air pollution as a serious problem in developing countries [13]. According to particle matter index (PMI); there are pollutants such as HC,Pb,No2,Co2,Co,O3 and particles with PM of less than 2.5 all having genotoxic and carcinogenic effects on the genome. If the pollutants concentration increased up to 100 we have air pollution; however, the level lower of 100 indicates healthy air. The quality control center of air pollution in Tehran has announced that the quality of air in Tehran is in a critical situation. Also this center determined four districts as the most pollutant districts: 6, 7, 11, and 12. As a result, we selected districts 6 and 7 to collect our samples. The quality control center of air pollution in Tehran has compared the level of air pollution. The comparison of the level of air pollution from March to October of the three successive years in Tehran is presented in Figure 1 [14]. Meantime, the quality control center of air pollution in Guilan has not reported any air pollution in the rural areas in Lahijan [15]. In this study we used classic cytogenetic methods for the investigation of chromosome aberration to detect the effect of air pollution on chromosomes and genomic stability.

## Materials and method

### Sampling

**Figure 2:**
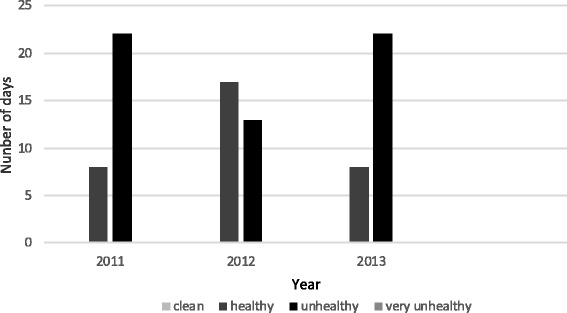
**Comparison of air quality index in October [** 14 **].**

**Figure 3:**
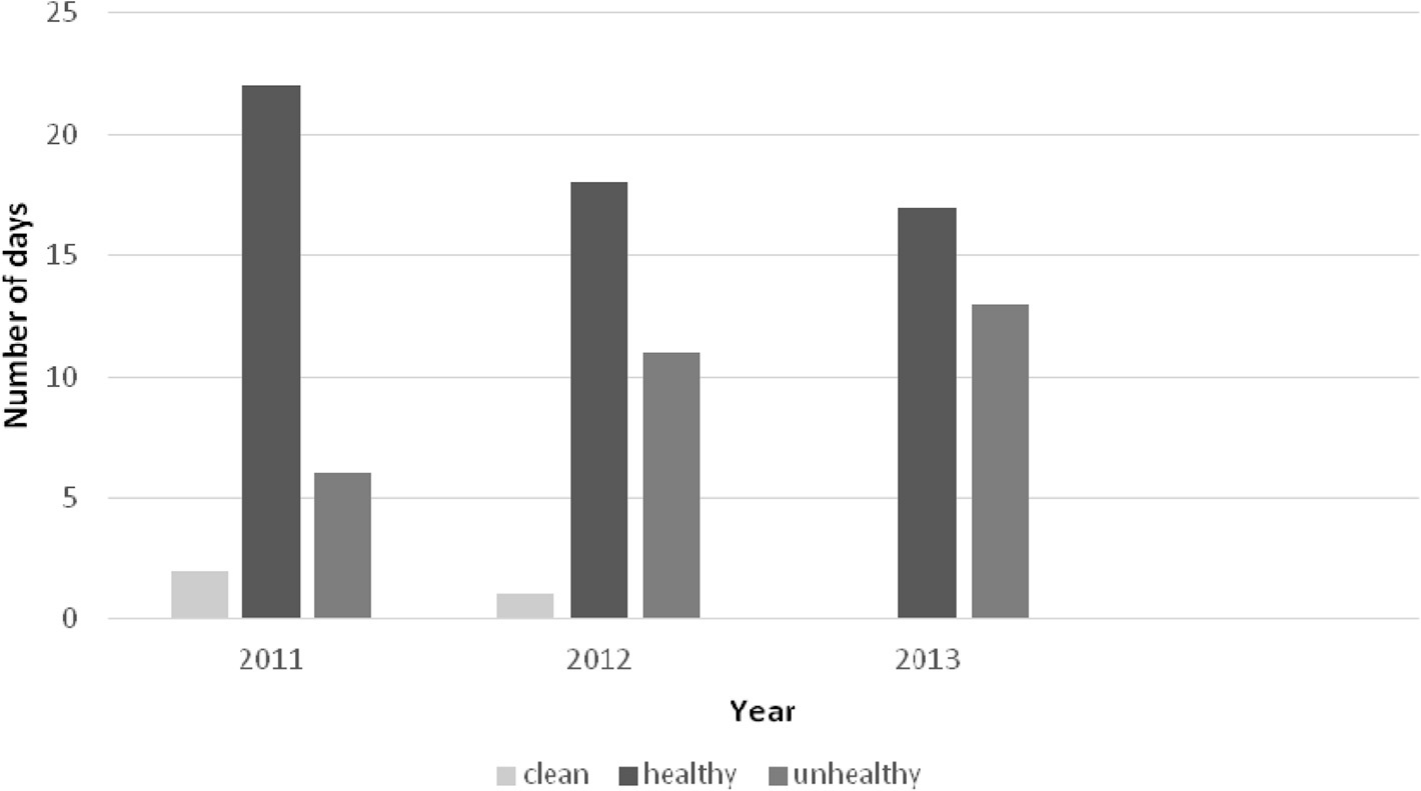
**Comparison of air quality index in November [** 14 **].**

The study population consisted of 30 taxi drivers (exposed group) from two polluted districts in Tehran and 30 taxi drivers from Lahijan (North of Iran) (the control group). The drivers’ working hours outdoor were eight hours daily. The exposed group consisted of 30 individuals, median age 35 years and the control group was 30 individuals, median age 33 years. All participating subjects were healthy volunteers who were recruited in convenience way (non-random sampling) and all completed a questionnaire. The inclusion criteria in this study were non smoker, no intake of alcohol, no family history of genetic disorder, non-exposure to chemical reagents and radiation. Any individual with medical treatment, radiation exposure or vaccination up to three months before sampling was not included in the study. The participants signed a consent form. The ethical code of practice of the University of Social welfare and Rehabilitation Sciences was observed. The sampling was performed during October and November 2013 for the case group and May to June for the control. The clean air was constant throughout the year for the control group. The level of air pollution during October and November in three successive years in Tehran is presented in Figures 2 and 3 [14].

### Cytogenetic technique

Heparinized blood was collected from the divers. Blood cultures were established within 24 h of specimen collection. Cell cultures were cultivated for 48 h at 37°C in RPMI 1640 with 0.5 cc 0f PBS and 1% phytohemagglutinin. Two duplicate cultures were set up from each sample. After 24 h we added 50 λ Colcemid to each tube and then after 1 h incubation both cultures were harvested using a classic technique that include centrifugation, treatment with hypotonic solution of KCL for 20 min followed by repeated fixation with methanol/acetic acid(3/1) [16].

### Microscopic analysis

After processing the cultures and preparation of slides, the slides were stained by Giemsa. The slides were examined with light microscope. A total of 100 chromosome spreads were examined for chromosome aberrations. The chromosome aberrations included chromatid gap, chromatid break, chromosome gap, chromosome break, chromosome rearrangements, ring, fragment, and dicentric chromosome. Gaps were scored as chromosome aberration. Dicentric and rearrangements were scored as two breaks. Ring, fragment, chromatid break, and chromosome break were scored as one break (Table 1). Chromosome breakage per cell was calculated for both the case and control groups.

**Table 1:** **Frequency of different types of chromosome aberration in case and control groups with aberrant cells (X** ^**2**^ **-test, P-value = 0.012)**

**abnormalities group**	**R**	**Dic**	**F**	**Ctg**	**Ctb**	**Csg**	**Csb**	**tr**	**qr**	**Rea**
Case	No	No	14 (26.7%)	15 (23.3%)	7 (20%)	1 (3.3%)	2 (6.7%)	No	No	No
control	NO	No	1 (3.3%)	4 (10%)	0 (0%)	1 (3.3%)	1 (3.3%)	No	No	No

R (Ring), Dic (Dicentric), F (Fragment), Ctg (Chromatid gap), Ctb (Chromatid break), Csg (Chromosome gap), Csb (Chromosome break), tr (triradial), qr (quadriradial), Rea(rearrangement).

### Statistical analysis

Data were analyzed using SPSS 11.5 software. Data are shown as frequency and percentage in study groups. X^2^test was carried out. P value of less than 0.05 was considered as statistically significant.

## Results

In this study, we have discovered an increased level of chromosome aberration in the drivers from polluted districts of Tehran. The difference was significant, using Chi-square test (p-value = 0.012) (Table 1). Based on the comparison of the level of chromosome breakage in case and control groups, there was an increased level of chromosome breakage (fragment, chromatid break, and chromosome break) in the drivers from polluted districts of Tehran, although not significant, using Fisher exact test (p-value = 0.300). The number of aberrant cells with chromosome aberration in 1000 scored cells showed a significant difference between the case and control groups, using X^2^-test ( P-Value = 0.001)(Table 2). Also there is no significant difference between the average age of two groups using t-test (p-value = 0.191).

**Table 2:** **Frequency of aberrant cells with breaks**

**Group**	**Aberrant No. of cells with breaks**	**Total No. of cells scored**	**Rate of aberrant cells/1000 (CI95%)**	**OR (CI95%)**	**P-Value**
Case	35	3000	1.17 (0.83-1.61)	5 (2.2-11.3)	0.001*
Control	7	3000	2.3 (7.02-1.62)		

OR: Odd Ratio, ( X^2^-test).

## Discussion

The air quality of Tehran has deteriorated in recent years. In particular during autumn and winter seasons the concentration of pollutants increase because of the inverted air phenomenon [14]. The effect of air pollution on morbidity and mortality has become a public health concern [17]. Numerous studies have related high levels of air pollutants to chromosome instability. Genomic instability is a principal agent that can lead to cancer [18]. Some studies have indicated that air pollution causes epigenetic alterations, mitochondrial and nuclear damage. Such genetic alterations can account for behavior disorder in Autistic children. These genetic changes are both microscopic and submicroscopic that can be detected using micro array analyses and DNA sequencing [19]. Similar studies have shown different panel of methylation in DNA in industrial residents exposed to pollutants in comparison with rural residents. They used polymerase chain reaction (PCR) and pyrosequencing to investigate these changes on template of DNA methylation with biomarkers such as LINE-1, P53, IL-6, and P16. Several of these methylation markers showed differences between industrial workers and rural controls [20]. Harmful effects of air pollution on health have been demonstrated in several investigations. It can lead to several diseases such as cancer, especially lung cancer. Stress oxidative damage results in inflammation and increasing coagulopathy activities in blood [21]. Several studies have used cytogenetic parameters including chromatid and chromosome aberrations to demonstrate the adverse effect of air pollutants on genomic stability. Both classic and molecular cytogenetic techniques have been used [22]. Few studies have compared conventional cytogenetic method with FISH and Micronucleus (MN) techniques and found noticeable differences between conventional cytogenetic and the latter techniques [23]. The FISH technique using whole chromosome painting probes is as a rapid and sensitive method for detecting structural rearrangements, in particular reciprocal translocations. Such rearrangements can be inherited and passed on to the next generation if affecting the germ cells. The micronucleus technique is also an efficient method for the detection of aneuploidies and large number of cells can be screened [24]. Using of FISH and Micronucleus (MN) can be useful to detect translocations and aneuploidies in the nuclei [25]. In some studies focusing on the different seasons, significant differences in chromosome aberrations have been found during summer and winter [26]. In a study by Rossner et al. using classic Cytogenetic technique, they determined the level of vitamins A, C, E, and folate in both case and control groups and did not find any significant difference between the two groups [27]. The level of chromosome aberration was not significant either.

In this study using classic Cytogenetic technique we demonstrated a significantly increased level of chromosome aberration (gaps and breaks) in the drivers from polluted districts of Tehran compared to drivers from non-polluted areas in North of Iran. However this is a preliminary study and such an investigation should be carried out on a larger sample size.

## Conclusion

An increased level of chromosome breakage was present in the drivers from polluted districts of Tehran compared to drivers from non-polluted areas in Lahijan. Based on these results and the importance of air pollution on morbidity and mortality, it is highly recommended that further similar studies on a larger sample size from other polluted areas of Tehran should be carried out for more conclusive results. Use of FISH and Micronucleus techniques are also highly recommended.

## References

[1] Yang W, Omaye ST (2009). Air pollutants, oxidative stress and human health. Mutat Res.

[2] Novotna B, Topinka J, Solansky I, Chvatalova I, Lnenickova Z, Sram RJ (2007). Impact of air pollution and genotype variability on DNA damage in prague policeman. Toxicol Lett.

[3] Domenico Franco M, Silvia A, Livia A, Harrie Besse L, Maria B, Nigel B (2013). Micronulei in cord blood lymphocytes and associations with biomarkers of exposure to carcinogens and hormonally active factors, gene polymorphisms and gene expression: The newgeneris cohort. ehp.

[4] Valavanidis A, Vlachogianni T, Fiotakis K, Loridas S (2013). Pulmonary Oxidative Stress, Inflammation and Cancer: Respirable Particulate Matter, Fibrous Dusts and Ozone as Major Causes of Lung Carcinogenesis through Reactive Oxygen species mechanisms. Int J Environ Res Public Health.

[5] Bocskay KA, Tang D, Orjuela MA, Liu X, Warburton DP, Perera FP (2005). Chromosomal aberrations in cord blood Are associated with prenatal exposure to carcinogenic polycyclic aromatic hydrocarbons. Cancer Epidemiol Biomarkers Prev.

[6] Moller P, Loft S (2010). Oxidative damage to DNA and lipids as biomarkers of exposure to air pollution. Environ Health Prospect.

[7] Hoeijmakers JHJ (2009). DNA damage, aging, and cancer. N Engl J Med.

[8] Rossner P, Rossnerova A, Spatova M, Beskid O, Uhlirova K (2012). Analysis of biomarkers in a Czech population part II: chromosomal aberrations and oxidative stress. Mutagenesis.

[9] **Julio Cesar Nepomuceno:** Nutrigenomics and cancer prevention. **2013, chapter 17, 1–26.**

[10] **Zhiyuan Shen, Robert Wood Johnson:** Genomic instability and cancer: an introduction**. 2011,** 3**:1–3.** 10.1093/jmcb/mjq057 21278445

[11] Negrini S, Gorgoulis VG, Halazonetis TD (2010). Genomic instability an evolving hallmark of cancer. Nature.

[12] Sram RJ, Beskid O, Binkova B, Rossner P, Smerhovsky Z (2004). Cytogenetic analysis using fluorescence in situ hybridization to evaluate occupational exposure carcinogens. Elsevier.

[13] Word Health Organization. Outdoor air quality database. Geneva: WHO;2011.available at: http://www.who.int/phe/health_topics/outdoorair.

[14] Air pollution data [http://air.tehran.ir] March 2013.

[15] Air pollution data [http://gilan.doe.air.ir] March 2013.

[16] Human Cytogenetics: In *A practical Approach Volume I.* Edited by Roony D, Czepulkowski BH. Oxford University press; 2001.

[17] Kleeberger SR (2003). Genetic aspects of susceptibility to air pollution. Eur Respir J.

[18] Demetrioul CA, Ole Raaschou N, Peter M, Roel V, Domenico P, Marc Chadeau H (2011). Biomarkers of ambient air pollution and lung cancer: a systematic review. Occup Environ Med.

[19] Moyra S (2014). Nuclear and Mitochondrial Genome defects in Autism. Genomic instability and Impact of epigenetic and environmental factors, Comprehensive Guide to Autism.

[20] Marco P, Valentina B, Armelle M, Petcharin S, Adisorn J, Suleeporn S: **DNA methylation differences in exposed workers and nearby residents of the Ma Ta Phut industrial estate, Rayong, Thailand.** *IJe* 2012**:**1–8. 10.1093/ije/dys129 PMC3535756 23064502

[21] Pedersen M, Vinzents P, Petersen JH, Kleinjans JCS, Plas G, Kirsch-Volders M, Dostál M, Rössner P (2006). Cytogenetic effects in children and mothers exposed to air pollution assessed by the frequency of micronuclei and fluorescence in situ hybridization (FISH): A family pilot study in the Czech Republic. Mutat Res.

[22] Xiangy M, Aoy L, Yang H, Liu W, Sun L, Han X, Liu J, Cao J (2012). Chromosomal damage and polymorphisms of metabolic genes among 1, 3-butadiene-exposed workers in a matched study in China. Mutagenesis.

[23] Kim Vande L, Eleni F, Ilse D, Georgia C, Maria K, Gina P (2011). Maternal and Gestational Factors and Micronucleus Frequencies in Umbilical Blood: The NewGeneris Rhea Cohort in Crete. Children’s Health.

[24] Sram RJ, Beskid O, Rössnerova A, Rössner P, Lnenickova Z, Milcova A, Solansky I, Binkova B (2007). Environmental exposure to carcinogenic polycyclic aromatic hydrocarbons; the interpretation of cytogenetic analysis by FISH. Toxicol Lett.

[25] Holland NT, Luoping Z, Smith MT: **Cytogenetic markers and air pollution, In relationship between acute and chronic effects of air pollution.** 2000**:**65–78.

[26] Demirgil GC, Erdem O, Gaga EO, Altug H, Van Doorn W, Burgaz S (2013). Cytogenetic biomonitoring of primery school children exposed to air pollutants:micronuclei analysis of buccal epithelial cells. Environ Sci Pollut Res.

[27] Rossner P, Bavorova H, Ocadlikova D, Svandova E, Sram RJ (2002). Chromosomal aberrations in peripheral lymphocytes of children as biomarkers of environmental exposure and life style. Toxicol Lett.

